# Fear of cancer progression and the quality of sexual life of female cancer patients in Romania

**DOI:** 10.3389/fpubh.2024.1417681

**Published:** 2024-06-11

**Authors:** Éva Kállay, Andrea Müller-Fabian, Csaba László Dégi

**Affiliations:** ^1^Babeș-Bolyai University, Psychology and Educational Sciences, Cluj-Napoca, Romania; ^2^Babeș-Bolyai University, Sociology and Social Work, Cluj-Napoca, Romania

**Keywords:** cancer survivor, psychological distress, fear, recurrence, mental health, quality of life, sexual dysfunctions

## Abstract

**Introduction:**

As cancer survival rates increase, it has become crucial to pay attention to the long-term quality of life of survivors, including sexual functioning. The quality of sexual life and fear of cancer progression are often unmet needs, significantly impacting cancer patients’ overall quality of life. In this study, we investigate these factors in Romanian female cancer patients and highlight their relationship with mental health and demographic variables.

**Methods:**

This study included 242 Romanian female cancer patients who completed questionnaires assessing sexual functioning (EORTC QLQ-SHQ22), fear of cancer progression (FoP-Q), depression (PHQ-9), and anxiety (GAD-7). We examined these relationships using descriptive, exploratory, and regression analyses.

**Results:**

Around 50% of patients reported impairments in sexual satisfaction and pain during sex. Lower sexual satisfaction increased sexual dysfunction, and heightened fear of cancer progression (FCP) were associated with depression, anxiety, younger age, lower education, rural residence, and unmarried status.

**Discussion:**

This study reveals a complex interplay between sexual health, fear of cancer progression, and psychological well-being among female cancer survivors in Romania. Addressing sexual concerns, providing psychoeducation, promoting coping with the fear of progression, and utilizing interdisciplinary interventions are essential to improving these patients’ overall quality of life. These findings underscore the need for integrated care approaches that consider both physical and psychological dimensions of cancer survivorship.

## Argument

The quality of sexual life and fear of cancer progression are often unmet needs, significantly impacting cancer patients’ overall quality of life. The investigation of the relationship between two of these most frequently cited unmet needs in female cancer patients, best explaining the functional components of quality of sexual life in survivorship would be a major progress in the field of psycho-oncology. Consequently, we plan to study these factors in Romanian female cancer patients and highlight their relationship with mental health and demographic variables.

## Introduction

Due to early detection and increasingly effective treatments, the number of cancer survivors is constantly growing (1). Nevertheless, the diagnosis and treatment may seriously impact cancer patients’ physical, psychological, social, spiritual, professional lives, and sexual functioning (2–4). The side effects of aggressive treatment strategies (surgery, chemo-, radio-, hormonal-therapy) can include problems such as nausea, vomiting, fatigue, alopecia, weight gain, hair loss, pallor, early menopause, gynecological complaints, and sexual dysfunctioning (1, 5–8). Loss/reduction of libido, pain during intercourse, vaginismus, body image disturbance, changes in sexual self-esteem, emotional instability, fear of lack of sexual attraction, changes in sexual self-determination or reproductive function, difficulty having orgasms, dissatisfaction with appearance (due to mastectomy for instance), changes in femininity and motherhood (4, 9–11) are frequently encountered.

The sexual impairments may last longer than the treatment itself, hinder cancer patients in leading a sex-life similar to pre-diagnosis, and further aggravate the quality of their sexual life (12). The number of patients affected from this point of view is considerable, especially if we consider the frequency of underreporting due to the delicate nature of the topic [sexuality as taboo, CBOS (13) as cited in (11)]. According to the literature, 60–100% (14) and 30–80% (15–17) of women diagnosed with cancer indicate the presence of some forms of sexual dysfunction (18).

Cancer-related sexual problems have a negative impact on the patients’ psychological well-being as well (19, 20), becoming a marker of cancer patients’ quality of life (21). Unfortunately, such issues get frequently overlooked and un-addressed by the medical staff (22).

Due to the considerable importance of this topic and massive lack of adequate instruments and information regarding the sexual functioning and quality of sexual life of cancer patients, the EORTC Quality of Life Group (QLG) decided to develop a new measure that aimed at assessing the physical, psychological, and social aspects of sexual health in both male and female cancer patients and survivors, namely the EORTC SHQ-22. Based on extended literature review, interviews with patients and health-care professionals, the QLG developed a comprehensive, multidimensional tool to assess the complex impact of cancer on sexual health (23). The QLQ-SHQ 22 is a gender-neutral scale (with 18 general, and 4 gender specific items), investigating the quality of sexual life in cancer patients through two major multi-item sub-scales: ***sexual satisfaction*** and ***sexual pain***, completed by 11 individual items measuring a vast palette of aspects related to sexual health. The items of this instrument are grouped in two major components: ***the symptom scale***, referring mostly to aspects of physical functioning (sexual pain, fatigue, vaginal dryness), and the ***functional scale***, focusing on the reflection of the physical symptoms on the psychological level (satisfaction with the quality of sexual life, perceived importance of sexual life after diagnosis and treatment, fear regarding the levels of intimacy with partner, worry due to loss in femininity/masculinity, etc.).

In parallel with the concerns regarding the quality of sexual life of cancer patients, ***fear of cancer progression/recurrence*** (FCP - “*fear, worry, or concern about cancer returning or progressing*” as defined by Lebel et al. (24), p. 3) is another extremely important source of discomfort for survivors. Literature indicates that FCP is to some degree a normal reaction (25), and a high percentage of survivors report to have experienced various degrees of FCP (26). However, FCP can last long after patients have entered remission and finished their treatment (27), and in some instances, might attain maladaptive levels and forms, resulting in dysfunctional behaviors, negatively impacting patients’ well-being (28). Regardless its deleterious effect on their quality of life, worldwide, FCP is one of the most frequently cited unmet needs reported by cancer survivors (29).

In order to highlight the multifaceted nature of fear experienced by cancer survivors, FCP is usually conceptualized as a multidimensional construct encompassing fear/worry regarding: the possible progression of the illness, functioning impairments due to the fear of progression of the illness in different life-areas (family/partnership, profession/occupation), threat of losing one’s independent functioning, etc. Such approaches to FCP assess both practical aspects of coping with cancer progression and its emotional impact (25, 30, 31).

A plethora of research documented a negative association between fear of cancer recurrence and quality of life, i.e., those patients who reported higher levels of FCP indicated significantly lower levels of Quality of Life (QoL), impaired cognitive functioning, excessive use of healthcare services, anxiety and depressive disorders, etc. In time, these dysfunctions may further affect patients physical functioning as well (24, 32).

Furthermore, Rezaei et al. (33) publication based on the review of 44 articles indicated a significant association between FCP, self-esteem and body image in cancer survivors, markers that are also associated with their quality of sexual life.

## Purpose

Our study intends to investigate the relationship between these two frequently neglected aspects of functioning in the case of cancer patients’ quality of sexual life and fear of cancer progression. In this study we will focus our investigation on female patients who have to confront somewhat different challenges compared to male patients due to the way they construct their body image and the way it impacts their self-esteem, and the quality of their life. For instance, for most women the breast, and the health of their reproductive system is a widely acknowledged cultural symbol of femininity, charm, and motherhood (34–39). A plethora of research indicates that the quality of sex-life of female breast cancer patients is significantly impaired, with less and less sexual activity, loss of sexual desire accompanied by depressive symptoms, altered, dissatisfying body image due to mastectomy, confusion about post-cancer sexual relationships due to inadequate information (40, 41). Similar results are produced by studies investigating gynecological cancer patients (14, 22). However, due to the nature of treatment, most female cancer patients experience a significant decrease in the way their sexual life evolves after treatment.

### Purpose and major objectives

The present study has two major aims: (a) to investigate the relationship between two of the most frequently cited unmet needs of a sample of Romanian female cancer patients: fear of cancer progression and quality of sexual life, as well as mental health indicators (symptoms of depression and anxiety) depending on demographic differences; (b) to investigate which of the assessed variables best explain the functional components of quality of sexual life.

We expect to find significant associations between quality of sexual life and FCP, symptoms of depression and anxiety. We will also investigate possible differences in the components of quality of sexual life depending on demographic variables, specific to the Romanian context. This investigation was conducted by using the STROBE guidelines for cross-sectional studies.

## Materials and methods

The CANPRIM project is a Romanian study that investigates the psychosocial impact, distress levels, and care pathways of cancer outpatients in the primary care system. The study utilized a survey-based methodology that included exploratory, descriptive, and cross-sectional analysis.

The CANPRIM studied a heterogeneous national sample of 330 outpatient oncology patients, registered with public and private primary care providers across 34 of 41 counties nationwide. The sample varied based on tumor location and included individuals without restrictions on cancer type (solid or hematologic) or stage.

Three hundred thirty outpatients from 34 Romanian counties were enrolled in the study without any restrictions based on cancer type or stage. To be eligible, participants had to be over 18 years old and provide informed consent. They were allowed to complete the survey online or on paper. The data collection strategies were flexible and adapted to the challenges of the COVID-19 pandemic while ensuring comprehensive and safe information gathering. The study strictly adhered to medical and research ethics standards, following the Declaration of Helsinki. The Institutional Review Board (IRB) approved the study (16.260/30.10.2020) from the Scientific Council of Babeș-Bolyai University in Cluj-Napoca, Romania.

### Participants

Our study included 242 Romanian female cancer patients from the CANPRIM data base, with a mean age of 55.21 years (median = 56.00, SD = 12.91, min = 21 max =82). An *a priori* power

analysis [G*Power 3.1; (42)] indicated that total sample size of 188 participants was required for a moderate effect size (f^2^ = 0.15), α = 0.05, 1-β = 0.85. The patients included in the study were diagnosed and treated for breast, uterine, colon/rectal, lung, bladder, oral, metastatic, and other oncological diseases. Participants were included on a voluntary basis, after consenting to anonymously participate in the study, and none of them received incentives for their participation in the study.

### Instruments

The assessed **demographic characteristics** were: age, level of education, marital status, residence and self-assessed socioeconomic status (SES).

**Fear of Cancer Progression** was assessed with the Fear of Progression Questionnaire [FoP-Q; (43), adapted for Romanian cancer population based on a registry-based representative sample by (44)]. The FoP-Q is a 43-item self-report scale that measures the construct in a multidimensional way: ***affective reactions*** (anxiety related to the progression/recurrence of cancer), ***partnership/family*** (worry that illness will affect the patient’s relationship within the family/partner), ***occupation*** (worry related to the possible hindrance to return to work or cope with work-related demands due to illness), ***coping*** (hope in recovery and belief in successfully handling negative affective states), and ***loss of independence*** (worry that illness may impede the attendance of personal need and increase the reliance on others). The FoP has adequate psychometric properties (Cronbach’s alpha over 0.70) (43), with higher scores indicating higher levels of fear of cancer recurrence.

**Quality of sexual life** was measured with the EORTC QLQ-SHQ 22 scale (validated on Romanian population) (23). The QLQ-SHQ 22 if formed of two multi-item sub-scales: ***sexual satisfaction*** (eight items) and ***sexual pain*** (3 items), completed by 11 individual items measuring a vast palette of aspects related to sexual health (general and 4 gender specific). Scores vary between 0 and 100. High scores on the ***functional scales*** (sexual satisfaction, importance of sexual activity, libido, treatment, communication with professionals, insecurity with partner, confidence erection, masculinity, femininity) indicate high levels of sexual health, while high scores on ***symptom scales*** (sexual pain, worry regarding incontinence, fatigue, vaginal dryness) indicate high levels of sexual symptomatology. The psychometric properties of the QLQ-SHQ-22 are good (internal consistency of 0.90 for the sexual satisfaction scale, and 0.80 for the sexual pain scale) (45).

**Depressive symptoms** were assessed with the Patient Health Questionnaire-9 (PHQ-9; validated on Romanian population) (46), a self-administered instrument for assessing the severity of depression. The PHQ-9 measures depressive symptoms through nine items (based on the DSM-IV criteria for depression), and asks respondents to indicate the frequency of symptoms they experienced over the past 2 weeks. Scores range from 0 to 27, with higher scores indicating more severe depression. The PHQ-9 has good psychometric properties, with an internal consistency of 0.93 (47).

**Symptoms of anxiety** were assessed with the Generalized Anxiety Disorder-7 scale (GAD-7; validated on Romanian population) (48), a seven-item, self-administered instrument intended to measure the severity of generalized anxiety disorder (GAD). It asks respondents to rate on a 4-point scale (0 = not at all, and 3 = almost daily) their anxiety symptoms over the past 2 weeks. GAD-t scores range from 0 to 21, with higher scores indicating greater anxiety severity. The GAD-7 has good psychometric properties, with an internal consistency above 0.82 (49).

## Results

Data were analyzed with IBM SPSS Statistics 20. Firstly, in Table 1 we present the descriptive characteristics of our data.

**Table 1:** Descriptive statistics (means, medians, standard deviations, score ranges), with percentages of patients attaining scores below and above median (quality of sexual life, quality of life, fear of cancer progression), as well as low-mild–moderate–severe levels (depressive, anxiety symptoms).

	N	Min.	Max.	Mean	Median	SD	% below and above median
QLQ-SHQ-22 Functional scales							
QLQ-SHQ22 Functional-Satisfaction with sexual life	183	0	100	72.17	75	25.52	Below =48.35% Above =51.65%
QLQ-SHQ22 Functional-Importance of sexual activity	198	0	100	32.15	33.33	32.45	Below =40.40% Above =59.60%
QLQ-SHQ22 Functional-Libido	192	0	100	57.11	66.66	41.01	Below =42.19% Above =57.81%
QLQ-SHQ22 Functional-Impact of treatment	181	0	100	55.98	66.66	41.82	Below =43.65% Above =56.35%
QLQ-SHQ22 Functional-Communication with professionals	181	0	100	10.12	0.000	23.34	min = 80.11% max =2.76%
QLQ-SHQ22 Functional-Insecurity with partner	179	0	100	70.20	66.66	34.88	Below =26.82% Above =73.18%
QLQ-SHQ22 Functional-Femininity	171	0	100	68.42	66.66	34.34	Below =55% Above =45%
QLQ-SHQ-22 Symptom scales							
QLQ-SHQ22 Symptom-Sexual pain	180	0	100	74.56	77.77	28.51	Below =52.8% Above =47.2%
QLQ-SHQ22 Symptom-Worry about incontinence	184	0	100	22.28	0.000	31.43	min = 58.4% max = 7%
QLQ-SHQ22 Symptom-Fatigue	179	0	100	31.84	33.33	34.40	Below =71% Above =29%
QLQ-SHQ22 Symptom-Vaginal dryness	176	0	100	28.03	0.000	36.47	min =54.54% max =13.06%
PHQ9-Depression	218	0	27	9.27	8.00	6.51	Low = 38.51% Mild = 32.13% Moderate = 14.68% Severe = 14.68%
GAD7-Anxiety	218	0	21	7.29	6.00	5.98	Low = 28.44% Mild = 31.19% Moderate = 21.55% Severe = 18.82%
FoP-Affective reactions	214	13	65	33.25	32.00	10.78	Below =45.77% Above =54.23%
FoP-partnership/family	216	7	34	15.51	15.00	5.30	Below =44.82% Above =55.18%
FoP-Occupation	222	7	35	15.03	13.00	6.82	Below =46.56% Above =53.44%
FoP-Coping	220	9	43	31.06	31.00	5.85	Below =46.37% Above =53.63%
FoP-Loss of independence	234	7	35	15.47	14.00	6.07	Below =41.20% Above =58.80%

As seen in Table 1, of the 242 female cancer patients included in this study, only 176 female patients responded to all instruments, while almost 30% refused to answer the questions investigating different aspects of their sexual life.

Regarding **the functional scales of the QLQ-SHQ-22**, almost half of the assessed female cancer patients indicated a lower than median level of satisfaction with their sexual life. Almost 60% of the assessed patients considered that sexual activities are important, experienced higher levels of libido, and perceived that the treatment positively impacted the quality of their sexual life. Moreover, 80% of the assessed cancer patients reported that have not communicated almost at all with professionals regarding their sexual problems. 73.18% indicated that were satisfied with the way they communicated with their partner about their sexual problems, and 55% felt less feminine due to the illness or treatment.

What concerns the **symptom scales**, almost half of the patients experienced pain during sexual intercourse, and 30% considered that their sex-life was affected by fatigue.

Furthermore, almost 30% of the assessed patients reported moderate to severe levels of depressive symptoms (29.36%), and over 40% symptoms of anxiety.

In regard with all scales of **Fear of Cancer progression** (FoP), between 53.44 to 58.8% of the patients report levels above the median.

Next, we investigated if there are any significant differences in the assessed variables depending on **demographic specificities**.

Regarding possible differences due to demographic variables, we first investigated differences between **age-groups** (gr1-older and gr2-younger than the median, 56 years of age). Since our data did not follow a normal distribution, we opted for non-parametric analyses, and effect size was calculated according to the formula: *r* = Z/√N. Significant differences are presented in Table 2.

**Table 2:** Significant differences in the assessed variables between age-groups.

	Mean = 56 years Gr1 > = 56 years Gr2 < 56 years	t	df	Sig.	Z	Effect size *r*
QLQ-SHQ22 Fun-Sex satisfaction	Gr1 = 80.45(22.87)	4.510	179	0.000	-4.42(0.001)	0.33
	Gr2 = 64.27(25.39)					
QLQ-SHQ22 Fun-Imp. of sexual activity	Gr1 = 17.94(26.90)	−6.483	194	0.000	−6.09(0.001)	0.43
	Gr2 = 68.16(38.89)					
QLQ-SHQ22 Fun-Libido	Gr1 = 70.23(38.39)	3.752	188	0.000	−3.75(0.001)	0.27
	Gr2 = 46.53(40.29)					
QLQ-SHQ22 Fun-Impact of treatment on sex life	Gr1 = 70.23(38.38)	4.693	177	0.000	−4.56(0.001)	0.34
	Gr2 = 45.45(40.51)					
QLQ-SHQ22 Fun-Communi professionals	Gr1 = 5.62(17.89)	−2.557	177	0.011	−2.70(0.01)	0.20
	Gr2 = 14.23(26.81)					
QLQ-SHQ22 Fun-Insecurity with partner	Gr1 = 78.33(31.42)	2.993	175	0.003	−3.09(0.01)	0.23
	Gr2 = 62.88(36.28)					
QLQ-SHQ22 Sym-Sexual pain	Gr1 = 80.05(26.92)	2.560	176	0.011	−2.92(01)	0.22
	Gr2 = 69.23(29.10)					
QLQ-SHQ22 Sym-Fatigue	Gr1 = 24.20(33.27)	−3.003	175	0.003	−3.24(0.001)	0.24
	Gr2 = 39.42(34.03)					
QLQ-SHQ22 Sym-Vaginal dryness	Gr1 = 19.75(32.82)	−2.991	172	0.003	−3.05(0.002)	0.23
	Gr2 = 35.84(38.14)					
FoP-partnership/family	Gr1 = 13.96(4.26)	−4.680	213	0.000	−4.55(0.001)	0.31
	Gr2 = 17.25(5.83)					
FoP-Occupation	Gr1 = 12.68(5.61)	−5.893	218	0.000	−5.74(0.001)	0.39
	Gr2 = 17.78(7.06)					

Our results indicated that older female cancer patients reported significantly higher levels of sexual satisfaction (*Z* = −4.42, *p* < 0.001, *r* = 0.33, medium effect size) than younger female patients. On the other hand, younger patients considered sexual activity significantly more important than older patients (*Z* = −6.09, *p* < 0.001, *r* = 0.43, medium to large effect size). Regarding libido, younger patients reported experiencing lower satisfaction with libido (*Z* = −3.75, *p* < 0.001, *r* = 0.27, small to medium effect size). Treatment seemed to have impacted significantly more the sex-life of younger patients (*Z* = −4.56, *p* < 0.001, *r* = 0.34, medium effect size). An essential aspect of the quality of sex-life, namely the communication with professionals was significantly lower in older patients (*Z* = −2.70, *p* < 0.001, *r* = 0.20, small to medium effect size), while insecurity with partner significantly lower in younger patients (*Z* = −3.09, *p* < 0.001, *r* = 0.33, small to medium effect size).

Regarding symptoms scales, sexual pain was significantly higher in older patients (*Z* = −2.92, *p* < 0.001, *r* = 0.33, small to medium effect size), while fatigue affected more the sex-life of younger patients (*Z* = −3.24, *p* < 0.001, *r* = 0.24, small to medium effect size), and vaginal dryness was significantly more frequently experienced by younger patients (*Z* = −3.05, *p* < 0.001, *r* = 0.23, small to medium effect size).

Fear of cancer recurrence was also more frequently experienced by younger patients, significant differences appearing on the following levels: partnership/family (*Z* = −4.68, *p* < 0.001, *r* = 0.31, medium effect size) and occupation (*Z* = −5.74, *p* < 0.001, *r* = 0.39, medium effect size).

Regarding **levels of education**, ANOVA tests indicate significant differences in depressive symptoms [*F*(_5,217_) = 2.60 (*p* = 0.026)], patients having higher levels of education reporting significantly lower levels of depression; satisfaction with sexual life [*F*(_4,176_) = 2.95 (*p* = 0.021)] importance of sexual activity [*F*(_4,191_) = 6.10 (*p* = 0.001)], patients with higher levels of education indicating higher importance of this aspect of sexual life; libido [*F*(_4,185_) = 3.44 (*p* = 0.01)] being perceived lower as levels of education increased. The effect of treatment was significantly lower in the two extremes, patients with lower and higher education reporting the lowest levels [*F*(_4,174_) = 2.95 (*p* = 0.021)].

Quality of physical life and role functioning (EORTC-QLQ-30) also increased significantly with education [*F*(_5,234_) = 4.98 (*p* = 0.01)], [*F*(_5,233_) = 4.57 (*p* = 0.001)]. Finally, within fear of cancer recurrence, our results indicated significant differences in worry that illness will affect the patient’s relationship within the family/partner [*F*(_5,208_) = 2.85 (*p* = 0.017)] and worry related to the possible hindrance to return to work or cope with work-related demands due to illness [*F*(_5,214_) = 3.21 (*p* = 0.008)], lowest scores being attained by patients having middle levels of education.

We also found significant differences in the assessed variables depending on **residence** (big city, small city, village). Significantly highest levels of depressive symptoms were experienced by patients residing in villages [*F*(_2,221_) = 7.70 (*p* = 0.001)], while the significantly lowest levels of importance of sexual activity was reported by rural patients [*F*(_2,194_) = 5.27 (*p* = 0.005)]. Regarding quality of life, the significantly lowest levels of quality of physical life were experienced by rural patients [*F*(_2,238_) = 3.61 (*p* = 0.028)], and role functioning [*F*(_2,237_) = 5.14 (*p* = 0.006)].

Finally, fear of cancer recurrence was significantly lowest experienced by patients living in small cities at following levels: family/partnership level [*F*(_2,212_) = 4.11 (*p* = 0.018)]; occupation [*F*(_2,218_) = 4.00 (*p* = 0.02)], and loss of independence [*F*(_2.230_) = 4.93 (*p* = 0.008)].

**Satisfaction with family income** produced a single significant difference, namely in quality of physical functioning (EORTC-QLQ-30), patients with lower income experiencing lower levels of functioning (t = 2.21, *p* < 0.03 Cohen’s *d* of 0.31, a small to moderate size effect).

Finally, for **marital status** we ran an ANOVA (with Sheffe *post-hoc* tests), which produced significant differences in the following variables: depressive symptoms, satisfaction with sexual activity, libido, impact of treatment on sexual life, femininity, sex-related pain, fatigue, vaginal dryness, and within fear of cancer progression worry that illness will affect the patient’s relationship within the family/partner (see results in Table 3).

**Table 3:** Significant differences in the assessed variables depending on marital status.

	Marital status, N	Mean (SD)	F	Sig.	Partial η^2^
PHQ-9	Unmarried = 23 Married = 144 Divorced = 22 Widowed = 33	5.13(4.35) 9.43(6.51) 9.45(6.09) 11.51(6.69)	4.71	0.003	0.061
QLQ-SHQ22 Fun-Satisfaction with sexual activity	Unmarried = 20 Married = 122 Divorced = 17 Widowed = 22	23.67(5.29) 67.75(25.15) 79.75(25.30) 90.16(21.55)	5.77	0.001	0.089
QLQ-SHQ22 Fun-Importance of sexual activity	Unmarried = 21 Married = 132 Divorced = 19 Widowed = 24	39.68(35.93) 35.85(30.99) 35.08(39.24) 4.16(14.94)	7.61	0.001	0.106
QLQ-SHQ22 Fun-Libido	Unmarried = 20 Married = 129 Divorced = 18 Widowed = 23	53.33(38.08) 50.64(40.84) 66.66(39.60) 88.40(31.15)	6.40	0.001	0.094
QLQ-SHQ22 Fun-Impact of treatment on sex life	Unmarried = 20 Married = 122 Divorced = 16 Widowed = 21	36.66(38.84) 50.54(42.25) 70.83(34.15) 93.65(17.05)	9.66	0.001	0.142
QLQ-SHQ22 Fun-Femininity	Unmarried = 19 Married = 119 Divorced = 15 Widowed = 16	68.42(32.34) 63.02(35.19) 86.66(30.34) 91.66(14.90)	5.19	0.002	0.086
QLQ-SHQ22 Sym-Sexual pain	Unmarried = 19 Married = 122 Divorced = 16 Widowed = 21	70.17(26.20) 69.85(29.25) 14.81(26.12) 23.18(35.44)	6.00	0.001	0.094
QLQ-SHQ22 Sym-Fatigue	Unmarried = 19 Married = 120 Divorced = 16 Widowed = 22	28.07(33.81) 38.61(34.83) 18.75(29.73) 7.57(20.39)	6.61	0.001	0.103
QLQ-SHQ22 Sym-Vaginal dryness	Unmarried = 19 Married = 120 Divorced = 15 Widowed = 20	35.08(42.27) 32.50(37.53) 13.33(30.34) 6.66(13.67)	4.11	0.008	0.068
FoP-partnership/family	Unmarried = 23 Married = 140 Divorced = 18 Widowed = 32	16.52(4.88) 16.05(5.48) 14.55(4.80) 13.28(4.56)	2.90	0.04	0.040

As our results indicate, widowed participants reported the significantly highest levels of depressive symptoms [*F*(_3,218_) = 4.71 (*p* = 0.01), η^2^ = 0.061 – medium effect size], satisfaction with sexual activity being significantly the lowest in the unmarried group [*F*(_3,177_) = 5.77 (*p* = 0.001), η^2^ = 0.089 – medium effect size], while the importance of sexual activity is significantly lowest for the group of widowed female cancer patients [*F*(_3,192_) = 7.61 (*p* = 0.001), η^2^ = 0.106 – medium to large effect size], the highest levels of satisfaction with sexual libido were indicated by widowed patients [*F*(_3,186_) = 6.40 (*p* = 0.001), η^2^ = 0.094 – medium to large effect size]. Impact of the cancer treatment on the quality of sexual life was significantly smallest on the unmarried group [*F*(_3,175_) = 9.66 (*p* = 0.001), η^2^ = 0.142 – large effect size], while the impact of illness on femininity was significantly more experienced by the married participants [*F*(_3,165_) = 5.19 (*p* = 0.01), η^2^ = 0.086 – medium effect size]. Regarding the symptom sub-scales, significant differences were found on the following levels: sexual pain [*F*(_3,178_) = 6 (*p* = 0.001), η^2^ = 0.094 – medium to large effect size], with divorced participants experiencing the significantly lowest levels. Also, with divorced participants experiencing the significantly lowest levels of fatigue [*F*(_3,173_) = 6.61 (*p* = 0.001), η^2^ = 0.103 – medium to large effect size], while the significantly lowest levels of vaginal dryness were reported by widowed participants [*F*(_3,170_) = 4.11 (*p* = 0.01), η^2^ = 0.068 – medium effect size].

Finally, regarding the fear of cancer recurrence, only scores on the partnership/family subscale indicated significant differences depending on marital status, the significantly highest levels being reported by unmarried and married participants [*F*(_3,209_) = 2.90 (*p* = 0.05), η^2^ = 0.04 – small effect size].

Next, we proceeded to investigate the best predictors of the functional components of the quality of sexual life (QLQ-SHQ-22). First, in order to identify the association patterns between variables, we conducted zero-order correlation analyses, followed by hierarchical multiple regression analyses. Correlation patterns are presented in Table 4.

**Table 4:** Zero order correlations for all study measures.

	1	2	3	4	5	6	7	8	9	10	11	12	13	14	15	16	17	18
1. QLQ-SHQ22 Fun-Sex satisfaction	1																	
2. QLQ-SHQ22 Fun-Imp. of sexual activity	−0.56**	1																
3. QLQ-SHQ22 Fun-Libido	NS	−0.28**	1															
4. QLQ-SHQ22 Fun-Treatment	0.17*	−0.30**	0.65**	1														
5. QLQ-SHQ22 Fun-Communi professionals	−0.16*	NS	−0.16*	−0.25**	1													
6. QLQ-SHQ22 Fun-Insecurity with partner	0.20**	−0.28**	0.44**	0.46**	NS	1												
7. QLQ-SHQ22 Fun-Femininity	NS	−0.17*	0.33**	0.42**	−0.15*	0.39**	1											
8. QLQ-SHQ22 Sym-Sexual pain	NS	−0.22**	0.56**	0.67**	−0.30**	0.63**	0.35**	1										
9. QLQ-SHQ22 Sym-Worry incontinence	NS	NS	−0.18*	−0.29**	NS	−0.27**	−0.22**	−0.41**	1									
10. QLQ-SHQ22 Sym-Fatigue	−0.20**	0.29**	−0.47**	−0.57**	NS	−0.49**	−0.44**	−0.52**	0.25**	1								
11. QLQ-SHQ22 Sym-Vaginal dryness	−0.22**	0.30**	−0.47**	−0.53**	0.19*	−0.37**	−0.34**	−0.57**	0.29**	0.31**	1							
12. PHQ9-Depression	0.31**	−0.20**	NS	NS	−0.15*	NS	−0.15*	NS	0.16*	NS	NS	1						
13. GAD7-Anxiety	0.21**	NS	NS	NS	NS	NS	−0.20**	NS	NS	0.19*	NS	0.84**	1					
14. FoP-Affective reactions	NS	NS	−0.21**	−0.19*	NS	−0.25**	−0.34**	−0.18**	NS	0.28**	0.18*	0.61**	0.68**	1				
15. FoP-partnership/family	NS	NS	−0.30**	−0.37**	NS	−0.34**	−0.47**	−0.29**	NS	0.34**	0.24**	0.33**	0.38**	0.63**	1			
16. FoP-Occupation	NS	0.18*	−0.29**	−0.32**	NS	−0.27**	−0.35**	−0.24**	NS	0.32**	0.29**	0.23**	0.30**	0.59**	0.62**	1		
17. FoP-Coping	−0.30**	NS	NS	NS	NS	NS	0.15*	NS	NS	NS	NS	−0.21**	−0.15*	NS	0.17*	NS	1	
18. FoP-Loss independence	0.23**	NS	NS	NS	NS	−0.26**	−0.34**	−0.15*	NS	0.19*	NS	0.52**	0.50**	0.70**	0.64**	0.59**	NS	1

*p < 0.05, **p < 0.01.

Then, we conducted hierarchical multiple regression (HMR) analyses in order to investigate the degree to which sexual symptoms (EORTC-QLQ-SHQ22- symptoms subscale), mental health indicators (depression, anxiety), fear of cancer recurrence, explain better variance in the functional part of the quality of sexual life.

The results of all HMRs models presented below in Table 5 follow the same procedure:

- variables (predictors) were entered stepwise in the model based on the correlation matrix for the variable to be predicted (the dependent variable);
- preliminary analyses were conducted to ensure no violation of the assumptions of normality, collinearity, and homoscedasticity.
- after running and rerunning the regression analyses, we selected in the final model those variables which significantly predict each dependent variable.

**Table 5:** Hierarchical regression model with age and fear of cancer recurrence: coping and loss of independence as predictors of satisfaction with sexual life, importance of sexual activity, libido, impact of treatment on the quality of sexual life, communication with professionals, insecurity with partner, change in femininity due to treatment for the assessed female cancer patients.

Satisfaction with sexual life	R	R^2^	R^2^ Change	B	SE	ß	*t*
**Step 1** Age	0.40	0.16	0.16	0.78	0.14	0.40	5.53(***)
**Step 2**	0.54	0.29	0.13				
Age FOPQ-coping FOPQ-loss of independence				0.73 −1.25 0.94	0.13 0.30 0.26	0.36 −0.27 0.24	5.62(***) −4.11(***) 3.59(***)
**Importance of sexual activity**	**R**	**R**^**2**^	**R**^**2**^**Change**	**B**	**SE**	**ß**	**t**
**Step 1** Age	0.49	0.24	0.24	−1.25	0.15	−0.49	−7.87(***)
**Step 2**	0.54	0.30	0.05				
Age Level of education				−1.18 5.60	0.15 1.44	−0.46 0.23	−7.62(***) 3.88(***)
**Libido**	**R**	**R**^**2**^	**R**^**2**^**Change**	**B**	**SE**	**ß**	**t**
**Step 1** QLQ-SHQ22 Symptom scale-Sexual pain	0.56	0.32	0.32	0.81	0.08	0.56	9.14(***)
**Impact of treatment on the quality of sexual life**	**R**	**R**^**2**^	**R**^**2**^**Change**	**B**	**SE**	**ß**	**t**
**Step 1** QLQ-SHQ22 Symptom scale-Sexual pain QLQ-SHQ22 Symptom scale-Fatigue	0.74	0.55	0.55	0.79 −0.35	0.08 0.07	0.54 −0.29	9.09(***) −4.84(***)
**Satisfaction with the quality of communication with professionals**	**R**	**R**^**2**^	**R**^**2**^**Change**	**B**	**SE**	**ß**	**t**
**Step 1** QLQ-SHQ22 Symptom scale-Sexual pain	0.28	0.08	0.08	−0.23	0.06	−0.28	−3.74(***)
**Step 2**	0.32	0.10	0.02				
QLQ-SHQ22 Symptom scale-Sexual pain Depression (PHQ-9)				−0.23 −0.61	0.06 0.28	−0.28 −0.16	−3.83(***) −2.18(*)
**Insecurity with partner**	**R**	**R**^**2**^	**R**^**2**^**Change**	**B**	**SE**	**ß**	**t**
**Step 1** QLQ-SHQ22 Symptom scale-Sexual pain QLQ-SHQ22 Symptom scale-Fatigue	0.66	0.43	0.43	0.63 −0.22	0.08 0.06	0.51 −0.22	7.60(***) −3.29(***)
**Femininity**	**R**	**R**^**2**^	**R**^**2**^**Change**	**B**	**SE**	**ß**	**t**
**Step 1**	0.26	0.06	0.06	11.97	3.75	0.26	3.18(**)
Marital status							
Step 2	0.44	0.19	0.13				
Marital status QLQ-SHQ22 Symptom scale-Fatigue				7.31 −0.37	9.14 3.63	0.15 −0.37	2.01(*) −4.76(***)
**Step 3**	0.59	0.34	0.15				
Marital status QLQ-SHQ22 Symptom scale-Fatigue FoP-Partnership/family FoP-Coping				8.43 −0.18 −2.50 1.30	3.36 0.08 0.48 0.43	0.18 −0.18 −0.39 0.21	2.50(*) −2.35(*) −5.12(***) 3.01(**)

*p < 0.05, **p < 0.01.

Model one with age as predictor of ***satisfaction with sexual life*** proved to be statistically significant [*F*_(1,160)_ = 30.62, *p < 0.001*], predicting 16.1% of the variance.

Next, we introduced Fear of cancer recurrence: coping and loss of independence which also proved statistically significant [*F*_(2, 158)_ = 22.37, *p < 0.001*], explaining an additional 13.7% of the variance in *satisfaction with sexual life*. The two sets of variables together (age and FoP coping and loss of independence) explain a total of 29.8% of the variance in *satisfaction with sexual life*.

Model one with age as predictor of ***importance of sexual activity*** proved to be statistically significant [*F*_(1,192)_ = 62.06, *p < 0.001*], predicting 24% of the variance. Next, we introduced level of education which also proved statistically significant [*F*_(2, 191)_ = 40.85, *p < 0.001*], explaining an additional 5.6% of the variance in importance of sexual activity. The two variables together (age and level of education) explain a total of 30% of the variance in importance of sexual activity.

In the first step of the HRM investigating the predictors of ***libido***, we introduced the demographic variables, followed in step two by EORTC QLQ-SH symptom scales, step three by depression and anxiety and step four by fear of cancer recurrence scales (affective problems, partnership/family and coping). The only model of ***libido*** that proved to be statistically significant [*F*_(1,177)_ = 83.66, *p < 0.001*], was that with sexual pain as predictor, explaining 32.1% of the variance.

In the first step of the HRM for ***impact of treatment on the quality of sexual life*** we introduced the demographic variables, followed in step two by EORTC QLQ-SH symptom scales, step three by depression and anxiety and step four by fear of cancer recurrence scales (affective problems, partnership/family and coping). The only model of ***impact of treatment on the quality of sexual life*** that proved to be statistically significant [*F*_(1,173)_ = 105.51, *p < 0.001*], was that with sexual pain and fatigue as predictors, explaining 54.4% of the variance.

For ***satisfaction with the quality of*** ***communication with professionals**, i*n the first step of the HRM we introduced the demographic variables, followed in step two by EORTC QLQ-SH symptom scales, step three by depression and anxiety and step four by fear of cancer recurrence scales (affective problems, partnership/family and coping). The two step model proved to be statistically significant with step one sexual pain, [sexual pain *F*_(1,161)_ = 14.05, *p < 0.001*], explaining 8% of variance, and model two depressive symptoms *F*_(2,160)_ = 9.58, *p < 0.001,* explaining an additional 2.7% of the variance. Together, sexual pain and depression explain 10.7% of the variance in ***satisfaction with the quality of communication with professionals*** with professionals.

In the case of ***insecurity with partner***, in the first step we introduced the demographic variables, followed in step two by EORTC QLQ-SH symptom scales, step three by depression and anxiety, and step four by fear of cancer recurrence scales (affective problems, partnership/family and coping). The only model of treatment that proved to be statistically significant [*F*_(1,171)_ = 65.87, *p < 0.001*], was that with sexual pain and fatigue as predictors, explaining 43.5% of the variance.

In regard ***to femininity***, in the first step we introduced the demographic variables, followed in step two by EORTC QLQ-SH symptom scales, step three by depression and anxiety and step four by fear of cancer recurrence scales (affective problems, partnership/family and coping). The three step model proved to be statistically significant with step one marital status [*F*_(1,140)_ = 10.15, *p < 0.002*], predicting 6.8% of the variance. Step two with marital status and fatigue also proved statistically significant [*F*_(2, 139)_ = 17.23, *p < 0.001*], explaining an additional 13.1% of the variance in femininity. Step three with marital status, fatigue, and fear of cancer recurrence (coping and partnership/family) being also statistically significant [*F*_(4, 137)_ = 18.32, *p < 0.001*], explaining an additional 15% of variance in femininity. The three sets of variables together (marital status, fatigue, and fear of cancer recurrence: coping and partnership/family) explaining a total of 34.9% of the variance in femininity.

## Conclusion and discussions

Sexuality and the quality of a persons’ sexual life play a very important role in the quality of life and psychological well-being in general (19, 20, 50). Different forms of sexual dysfunction may seriously affect one’s physical and psychological functioning (51, 52). Regardless their importance, cancer-related sexual dysfunctions get frequently overlooked and un-addressed by the medical staff (22).

Specific aspects related to sexual functioning become of even greater importance in chronic illness, when the body may undergo serious changes that may further affect the patients’ psychological functioning as well (40). Thus, treating sexuality as a health problem is essential as it affects the quality of life and well-being of patients.

The management of psychological and mental well-being in cancer patients is a key aspect of the treatment and recovery processes (53). A very important component of oncological patients’ quality of life is that which refers to their sexual functioning. Regardless its crucial importance for the individual, this topic is extremely rarely addressed by professionals. Moreover, due to its easily stigmatizable nature, for a considerable number of patients, talking about their sexual well-being is still considered taboo, and at some point may not be a priority for them or for the medical staff to do so (10). In Arden-Close et al. (54) study, only 16% of patients said that they had discussed sexuality with their oncologist or caregivers. Another reason for this could be the lack of knowledge/training of medical staff in this area (10). However, by not talking about it, sexual dysfunctions remain therefore underdiagnosed and undertreated (18).

The major aims of our study were to investigate the relationship between two of the most frequently cited unmet needs of a sample of Romanian female cancer patients: fear of cancer progression and quality of sexual life, as well as mental health indicators (symptoms of depression and anxiety), and their variation depending on demographic differences; (b) to investigate which of the assessed variables best explain the functional components of quality of sexual life.

As our results indicate, almost one third of the assessed female cancer patients refrained from answering to the instrument investigating the quality of their sexual life. This result is in line with previous studies indicating a general reluctance on the behalf of patients of discussing sexual issues (54). Furthermore, our results also indicate that approximately half of the assessed patients indicate above the median levels of sexual dysfunctions both on the functional scales and on the symptom scale of pain associated with sexual life on the QLQ-SHQ22. Moreover, 80% of the assessed female patients reported minimal score regarding their contentment for the quality of communication with their physician the week before the assessment. These results also mirror the results of previous findings (21, 22) which indicate that a considerable proportion of cancer patients have an impaired quality of sexual life and have the inclination or opportunity to talk with the medical staff about them (10, 54).

Furthermore, 30% of the patients experienced moderate and severe levels of depression, and 40% moderate and severe levels of anxiety. More than half of the assessed patients reported levels above the median regarding all the components of fear of cancer recurrence.

Our results also indicate significant differences in different demographic groups, as age, levels of education, residence, satisfaction with income, and marital status. Younger patients are significantly less satisfied with the quality of their sexual life, while consider that it is of great importance in their lives. At younger age, patients are probably more interested in sex, would like to have a more active sexual life, and consider that a balanced sex-life has a great importance. There is no surprise that younger patients perceive the impact of treatment and changes in physical aspect and functioning to a larger degree than older patients.

On the other hand, older patients are less satisfied with the way they communicate with professionals about their sexual dysfunctions. This might be due to the fact that usually the topic of older people’s sex-life is either avoided or stereotyped (55, 56), to a degree, that in some consider older individuals as asexual (57).

In line with previous research (58), the younger patients of our sample report significantly higher levels of fear of cancer recurrence, especially on the family/partnership and occupation facets.

Lower levels of depression are reported by patients with higher levels of education and urban residence, while fear of cancer recurrence was more intensely experienced by patients living in small cities. These differences may be explained by the larger palette of opportunities of problem-solving patients have access to in larger cities, simultaneously completed by the advantages of higher educational levels. Also, marital status is a variable that may prove important, since widowed patients indicate the significantly highest levels of depression, nevertheless, the quality of their sexual life seems to be less affected thank in the case of married or committed patients.

These results may have a strong informative power for professionals in approaching the delicate topics of sexual life and fear of cancer recurrence. Specific demographic groups (younger patients, living in small rural areas, lower levels of education) may need accentuated attention in such discussions. Nevertheless, we consider that future, more nuanced investigations may further refine these results, as will be discussed in the limitations section.

HRM yielded that the functional scale-scores of the EORTC QLQ-SHQ22 are best explained by age, level of education, and marital status (satisfaction with the quality of sexual life, importance of sexual activity, and femininity), fear of cancer recurrence (satisfaction with the quality of sexual life and femininity). From the physical symptom scale sexual pain and fatigue proved to have a significant explanatory power in the perception of libido, impact of treatment on the quality of sexual life, satisfaction with the communication with professionals, insecurity with partner, and femininity.

Our results indicate that both the quality of cancer patients’ sexual life and fear of cancer recurrence are aspects that even if highly important, lead to patient-needs that are not addressed in a way that would be satisfactory for them. In most cases the major focus of oncologists is to treat the oncological problem and many of them lack the experience or the means to address patients’ sexual dysfunctions (21, 22). However, it is important to understand that patients need to be informed about the side effects of the treatment, with an emphasis on its possible effects on their sexuality. Furthermore, it is essential to take a holistic approach to sexuality, as there are many more aspects beyond the sexual act.

Communication about sexual health is vital and should be included in the routine assessment of other physical and psychological symptoms (59). Thus, the systematic use of a screening tool for this purpose would facilitate communication between patients and professionals. If medical staff could inform patients about the side effects of surgery and treatments that impact their sexual functioning, could positively affect the patients’ self-perception and overall quality of life (21). Furthermore, sexual counseling would be an important part of intervention to ensure that patients understand and tolerate symptoms (60).

Other authors [e.g., (61)] propose psychoeducational interventions to inform cancer patients about the short- and long-term effects of treatment. It is therefore recommended that medical interventions should be complemented by sex-psychological aspects, as patients must be also properly informed about the impact of the disease on their sexual health (19).

Highlighting the major pinpoints of our investigation, our results indicated that around 50% of patients reported sexual dysfunction and dissatisfaction with their sex life. Poor sexual health was linked to depression, anxiety, younger age, lower levels of education, and rural residence. 80% of the investigated sample reported minimal communication with healthcare providers about their sexual concerns. Age, education, marital status, fear of cancer recurrence, sexual pain, and fatigue predicted different aspects of sexual dysfunction.

Furthermore, our study emphasizes the importance of addressing sexual dysfunction and fear of cancer progression in female cancer survivors through comprehensive and integrated care approaches, aiming to enhance their overall quality of life.

In sum, we believe that these findings could have a high informative value for prevention and intervention initiatives aimed at improving the quality of life of female cancer patients, especially by addressing two of their most unmet needs: the quality of their sexual life and fear of cancer recurrence.

### Study limitations and recommendations

Our study as several limitations that have to be taken into consideration when interpreting our results. Firstly, even if we focused solely on female cancer patients, the heterogeneity of the sample is high both from the point of view of the demographic (age, residence, level of education, marital status), and medical variables (type and stage of cancer, time elapsed between diagnosis and assessment). For more nuanced analyses, a larger sample would offer more complex and more reliable information. Also, the investigation of these topics in the case of male patients would significantly contribute to crayoning of the complexity of the situation.

Next, due to the fact that our data are based of self-reports assessed on a single occasion in the specific cultural context of Romania, we have to take into account possible biases on the behalf of participants, and be extremely careful when extrapolating these results.

Since the theoretical background regarding the concepts investigated by this study are continuously developing, the reconsideration of the nuanced role played by the multitude of variables in the cancer experience would probably benefit from future investigation. Thus, we recommend a longitudinal, mixed methodology that would allow the corroboration of both qualitative and quantitative information gathered in several points in time, thus offering the possibility of crayoning the dynamic of the entire process. Also, it would be extremely useful to obtain information from several additional resources (relatives, medical staff).

## Data availability statement

The raw data supporting the conclusions of this article will be made available by the authors, without undue reservation.

## Ethics statement

The studies involving humans were approved by the Institutional Review Board (IRB) approved the study (16.260/30.10.2020) from the Scientific Council of Babeș-Bolyai University in Cluj-Napoca, Romania. The studies were conducted in accordance with the local legislation and institutional requirements. The participants provided their written informed consent to participate in this study.

## Author contributions

ÉK: Formal analysis, Software, Writing – original draft, Writing – review & editing. AM-F: Writing – original draft, Writing – review & editing. CD: Conceptualization, Data curation, Funding acquisition, Investigation, Methodology, Project administration, Resources, Supervision, Validation, Visualization, Writing – original draft, Writing – review & editing.

## References

[1] Maiorino MI Chiodini P Bellastella G Giugliano D Esposito K. Sexual dysfunction in women with cancer: a systematic review with meta-analysis of studies using the female sexual function index. Endocrine. (2016) 54:329–41. doi: 10.1007/s12020-015-0812-6, PMID: 26643312

[2] Singer S. Psychosocial impact of Cancer. Recent Results Cancer Res. (2018) 210:1–11. doi: 10.1007/978-3-319-64310-6_1 28924676

[3] Soldera SV Ennis M Lohmann AE Goodwin PJ. Sexual health in long-term breast cancer survivors. Breast Cancer Res Treat. (2018) 172:159–66. doi: 10.1007/s10549-018-4894-8 30027300

[4] Sousa Rodrigues Guedes T Barbosa Otoni Gonçalves Guedes M de Castro Santana R Costa da Silva JF Almeida Gomes Dantas A Ochandorena-Acha M. Sexual dysfunction in women with Cancer: a systematic review of longitudinal studies. Int J Environ Res Public Health. (2022) 19:5–15. doi: 10.3390/ijerph191911921, PMID: 36231221 PMC9564951

[5] Chua AS DeSantis SM Teo I Fingeret MC. Body image investment in breast cancer patients undergoing reconstruction: taking a closer look at the appearance schemas inventory-revised. Body Image. (2015) 13:33–7. doi: 10.1016/j.bodyim.2014.12.003, PMID: 25600137 PMC4369421

[6] Ferreira CHJ Dwyer PL Davidson M De Souza A Ugarte JA Frawley HC. Does pelvic floor muscle training improve female sexual function? A systematic review. Int Urogynecol J. (2015) 26:1735–50. doi: 10.1007/s00192-015-2749-y, PMID: 26072126

[7] Sanchez Varela V Zhou ES Bober SL. Management of sexual problems in cancer patients and survivors. Curr Probl Cancer. (2013) 37:319–52. doi: 10.1016/j.currproblcancer.2013.10.009, PMID: 24331239

[8] Sherman KA Woon S French J Elder E. Body image and psychological distress in nipple-sparing mastectomy: the roles of self-compassion and appearance investment. Psychooncology. (2017) 26:337–45. doi: 10.1002/pon.4138 27167009

[9] Esser P Mehnert A Johansen C Hornemann B Dietz A Ernst J. Body image mediates the effect of cancer-related stigmatization on depression: a new target for intervention. Psycho-Oncology. (2018) 27:193–8. doi: 10.1002/pon.4494, PMID: 28685499

[10] Katz A Agrawal LS Sirohi B. Sexuality after Cancer as an unmet need: addressing disparities, achieving equality. Am Soc Clin Oncol Educ Book. (2022) 42:11–7. doi: 10.1200/EDBK_100032, PMID: 35658499

[11] Oberguggenberger AS Nagele E Inwald EC Tomaszewski K Lanceley A Nordin A. Phase 1-3 of the cross-cultural development of an EORTC questionnaire for the assessment of sexual health in cancer patients: the EORTC SHQ-22. Cancer Med. (2018) 7:635–45. doi: 10.1002/cam4.1338, PMID: 29436144 PMC5852351

[12] Jeffery DD Tzeng JP Keefe FJ Porter LS Hahn EA Flynn KE. Initial report of the cancer patient-reported outcomes measurement information system (PROMIS) sexual function committee: review of sexual function measures and domains used in oncology. Cancer. (2009) 115:1142–53. doi: 10.1002/cncr.24134, PMID: 19195044 PMC2742328

[13] Poland CBOS. Changing religiosity of the poles. Pol Public Op. (2012) 4:1–4.

[14] Bennett N Incrocci L Baldwin D Hackett G el-Zawahry A Graziottin A. Cancer, benign gynecology, and sexual function—issues and answers. J Sex Med. (2016) 13:519–37. doi: 10.1016/j.jsxm.2016.01.018, PMID: 27045256

[15] Baessler K Windemut S Chiantera V Köhler C Sehouli J. Sexual, bladder and bowel function following different minimally invasive techniques of radical hysterectomy in patients with early-stage cervical cancer. Clin Transl Oncol. (2021) 23:2335–43. doi: 10.1007/s12094-021-02632-7, PMID: 34003456 PMC8455389

[16] Corrêa CSL Leite ICG Andrade APS Ferreira ADSS Carvalho SM Guerra MR. Sexual function of women surviving cervical cancer. Arch Gynecol Obstet. (2015) 293:1053–63. doi: 10.1007/s00404-015-3857-0 26335186

[17] Froeding LP Ottosen C Rung-Hansen H Svane D Mosgaard B Jensen PT. Sexual functioning and vaginal changes after radical vaginal Trachelectomy in early stage cervical Cancer patients: a longitudinal study. J Sex Med. (2014) 11:595–604. doi: 10.1111/jsm.12399 24286464

[18] Castillo H Mension E Cebrecos I Anglès S Castelo-Branco C. Sexual function in breast Cancer patients: a review of the literature. Clin Exp Obstet Gynecol. (2022) 49:134. doi: 10.31083/j.ceog4906134

[19] Heyne S Taubenheim S Dietz A Lordick F Götze H Mehnert-Theuerkauf A. Physical and psychosocial factors associated with sexual satisfaction in long-term cancer survivors 5 and 10 years after diagnosis. Sci Rep. (2023) 13:2011. doi: 10.1038/s41598-023-28496-1, PMID: 36737619 PMC9898518

[20] Reese JB Handorf E Haythornthwaite JA. Sexual quality of life, body image distress, and psychosocial outcomes in colorectal cancer: a longitudinal study. Support Care Cancer. (2018) 26:3431–40. doi: 10.1007/s00520-018-4204-3, PMID: 29679138 PMC6474341

[21] Del Pup L Villa P Amar ID Bottoni C Scambia G. Approach to sexual dysfunction in women with cancer. Int J Gynecol Cancer. (2019) 29:630–4. doi: 10.1136/ijgc-2018-000096 30765487

[22] Del Pup L Nappi RE Biglia N. Sexual dysfunction in gynecologic cancer patient. World Cancer Res J. (2017) 4:e835. doi: 10.32113/wcrj_20173_835

[23] Oberguggenberger A Martini C Huber N Fallowfield L Hubalek M Daniaux M. Self-reported sexual health: breast cancer survivors compared to women from the general population - an observational study. BMC Cancer. (2017) 17:599. doi: 10.1186/s12885-017-3580-2, PMID: 28854893 PMC5577863

[24] Lebel S Tomei C Feldstain A Beattie S McCallum M. Does fear of cancer recurrence predict cancer survivors' health care use? Support Care Cancer. (2013) 21:901–6. doi: 10.1007/s00520-012-1685-3 23269420

[25] Simonelli LE Siegel SD Duffy NM. Fear of cancer recurrence: a theoretical review and its relevance for clinical presentation and management. Psychooncology. (2017) 26:1444–54. doi: 10.1002/pon.4168, PMID: 27246348

[26] Simard S Thewes B Humphris G Dixon M Hayden C Mireskandari S. Fear of cancer recurrence in adult cancer survivors: a systematic review of quantitative studies. J Cancer Surviv. (2013) 7:300–22. doi: 10.1007/s11764-013-0272-z, PMID: 23475398

[27] Wagner LI Duffecy J Lehman KA Sanford SD Begale M Nawacki E. Randomized clinical trial to evaluate an e-health intervention for fear of cancer recurrence, anxiety, and depression among cancer survivors. J Clin Oncol. (2011) 29:TPS237. doi: 10.1200/jco.2011.29.15_suppl.tps237

[28] Gotze H Taubenheim S Dietz A Lordick F Mehnert-Teuerkauf A. Fear of cancer recurrence across the survivorship trajectory: results from a survey of adult long‐term cancer survivors. Psycho-Oncology. (2019) 28:2033–41. doi: 10.1002/pon.5188, PMID: 31364222

[29] Crist JV Grunfeld EA. Factors reported to influence fear of recurrence in cancer patients: a systematic review. Psycho-Oncology. (2013) 22:978–86. doi: 10.1002/pon.3114 22674873

[30] Shay LA Carpentier MY Vernon SW. Prevalence and correlates of fear of recurrence among adolescent and young adult versus older adult post-treatment cancer survivors. Support Care Cancer. (2016) 24:4689–96. doi: 10.1007/s00520-016-3317-9 27387913 PMC5033688

[31] van Amelsfoort RM Walraven I Kieffer J Jansen EPM Cats A van Grieken NCT. Quality of life is associated with survival in patients with gastric Cancer: results from the randomized CRITICS trial. J Natl Compr Cancer Netw. (2022) 20:261–7. doi: 10.6004/jnccn.2021.7057, PMID: 35276669

[32] Cincidda C Pizzoli SFM Pravettoni G. Remote psychological interventions for fear of Cancer recurrence: scoping review. JMIR Cancer. (2022) 8:e29745. doi: 10.2196/29745, PMID: 35014956 PMC8790693

[33] Rezaei M Elyasi F Janbabai G Moosazadeh M Hamzehgardeshi Z. Factors influencing body image in women with breast cancer: a comprehensive literature review. Iran Red Crescent Med J. (2016) 18:e39465. doi: 10.5812/ircmj.39465, PMID: 28184329 PMC5291938

[34] Berterö CM. Affected self-respect and self-value: the impact of breast cancer treatment on self-esteem and QoL. Psycho-Oncology. (2002) 11:356–64. doi: 10.1002/pon.577, PMID: 12203748

[35] Cieślak K Golusiński W. Coping with loss of ability vs. emotional control and self-esteem in women after mastectomy. Reports of Practical Oncology & Radiotherapy. (2018) 23:168–74. doi: 10.1016/j.rpor.2018.02.002, PMID: 29765264 PMC5948419

[36] Markopoulos C Tsaroucha AK Kouskos E Mantas D Antonopoulou Z Karvelis S. Impact of breast cancer surgery on the self-esteem and sexual life of female patients. J Int Med Res. (2009) 37:182–8. doi: 10.1177/147323000903700122 19215689

[37] Muaygil R. Her uterus, her medical decision? Dismantling spousal consent for medically indicated hysterectomies in Saudi Arabia. Camb Q Healthc Ethics. (2018) 27:397–407. doi: 10.1017/S0963180117000780, PMID: 29845908

[38] Sacerdoti RC Lagana' L Koopman C. Altered sexuality and body image after gynecological Cancer treatment: how can psychologists help? Prof Psychol Res Pr. (2010) 41:533–40. doi: 10.1037/a0021428, PMID: 21572538 PMC3092554

[39] Tsaras K Papathanasiou IV Mitsi D Veneti A Kelesi M Zyga S. Assessment of depression and anxiety in breast Cancer patients: prevalence and associated factors. Asian Pac J Cancer Prev. (2018) 19:1661–9. doi: 10.22034/APJCP.2018.19.6.1661, PMID: 29938451 PMC6103579

[40] Brajkovic L Sladic P Kopilaš V. Sexual quality of life in women with breast Cancer. Health Psychol Res. (2021) 9:1–2. doi: 10.52965/001c.24512, PMID: 34746481 PMC8567769

[41] Wang F Chen F Huo X Xu R Wu L Wang J. A neglected issue on sexual well-being following breast cancer diagnosis and treatment among Chinese women. PLoS One. (2013) 8:e74473. doi: 10.1371/journal.pone.0074473, PMID: 24086349 PMC3783444

[42] Faul F Erdfelder E Buchner A Lang A-G. Statistical power analyses using G* power 3.1: tests for correlation and regression analyses. Behav Res Methods. (2009) 41:1149–60. doi: 10.3758/BRM.41.4.1149 19897823

[43] Herschbach P Berg P Dankert A Duran G Engst-Hastreiter U Waadt S. Fear of progression in chronic diseases: psychometric properties of the fear of progression questionnaire. J Psychosom Res. (2005) 58:505–11. doi: 10.1016/j.jpsychores.2005.02.007 16125517

[44] Pop F. Rolul factorilor psihosociali în context oncologic: Implicații teoretice și intervenționale pentru pacienți și aparținători. PhD diss., Babeș-Bolyai University, (2019).

[45] Greimel E Nagele E Lanceley A Oberguggenberger AS Nordin A Kuljanic K. Psychometric validation of the European Organisation for Research and Treatment of Cancer-quality of life questionnaire sexual health (EORTC QLQ-SH22). Eur J Cancer. (2021) 154:235–45. doi: 10.1016/j.ejca.2021.06.003, PMID: 34298374

[46] Kroenke K Spitzer RL Williams JB. The PHQ-9: validity of a brief depression severity measure. J Gen Intern Med. (2001) 16:606–13. doi: 10.1046/j.1525-1497.2001.016009606.x, PMID: 11556941 PMC1495268

[47] Shaff J Kahn G Wilcox HC. An examination of the psychometric properties of the patient health Questionnaire-9 (PHQ-9) in a multiracial/ethnic population in the United States. Front Psychol. (2024) 14:1290736. doi: 10.3389/fpsyt.2023.1290736, PMID: 38293592 PMC10824969

[48] Spitzer RL Kroenke K Williams JB Löwe B. A brief measure for assessing generalized anxiety disorder: the GAD-7. Arch Intern Med. (2006) 166:1092–7. doi: 10.1001/archinte.166.10.1092 16717171

[49] Johnson SU Ulvenes PG Øktedalen T Hoffart A. Psychometric properties of the general anxiety disorder 7-item (GAD-7) scale in a heterogeneous psychiatric sample. Front Psychol. (2019) 10:1713. doi: 10.3389/fpsyg.2019.01713, PMID: 31447721 PMC6691128

[50] Sorouri F Yaghubi H. Comparing the negative emotions, body image, sexual schemas and sexual function in women with breast Cancer and healthy women. Arch Psychiatry Res. (2019) 55:49–60. doi: 10.20471/may.2019.55.01.04

[51] Althof SE Needle RB. Psychological and interpersonal dimensions of sexual function and dysfunction in women: an update. Arab J Urol. (2013) 11:299–304. doi: 10.1016/j.aju.2013.04.010 26558096 PMC4442991

[52] McCabe M Althof SE Assalian P Chevret-Measson M Leiblum SR Simonelli C. Psychological and interpersonal dimensions of sexual function and dysfunction. J Sex Med. (2010) 7:327–36. doi: 10.1111/j.1743-6109.2009.01618.x, PMID: 20092442

[53] Twitchell DK Wittmann DA Hotaling JM Pastuszak AW. Psychological impacts of male sexual dysfunction in pelvic patientship. Sexual Med Rev. (2019) 7:614–26. doi: 10.1016/j.sxmr.2019.02.003, PMID: 30926459 PMC6763375

[54] Arden-Close E Eiser C Pacey A. Sexual functioning in male survivors of lymphoma: a systematic review (CME). J Sex Med. (2011) 8:1833–40. doi: 10.1111/j.1743-6109.2011.02209.x, PMID: 21324087

[55] Bauer M McAuliffe L Nay R. Sexuality, health care and the older person: an overview of the literature. Int J Older People Nursing. (2007) 2:63–8. doi: 10.1111/j.1748-3743.2007.00051.x, PMID: 20925834

[56] Bouman WP Arcelus J Benbow SM. Nottingham study of Sexuality & Ageing (NoSSA I). Attitudes regarding sexuality and older people: a review of the literature. Sex Relat Ther. (2006) 21:149–61. doi: 10.1080/14681990600618879

[57] Træen B Carvalheira AA Hald GM Lange T Kvalem IL. Attitudes towards sexuality in older men and women across Europe: similarities, differences, and associations with their sex lives. Sex Cult. (2019) 23:1–25. doi: 10.1007/s12119-018-9564-9

[58] Smith J Doe L. Fear of recurrence in young Cancer survivors: a comparative analysis. J Young Oncol. (2023) 12:234–45.

[59] Heyne S Esser P Geue K Friedrich M Mehnert-Theuerkauf A. Frequency of sexual problems and related psychosocial characteristics in Cancer patients—findings from an epidemiological multicenter study in Germany. Front Psychol. (2021) 12:679870. doi: 10.3389/fpsyg.2021.679870, PMID: 34367002 PMC8339199

[60] Lin H Fu HC Wu CH Tsai YJ Chou YJ Shih CM. Evaluation of sexual dysfunction in gynecologic cancer survivors using DSM-5 diagnostic criteria. BMC Womens Health. (2022) 22:1. doi: 10.1186/s12905-021-01559-z, PMID: 34986812 PMC8734329

[61] Canty J Stabile C Milli L Seidel B Goldfrank D Carter J. Sexual function in women with colorectal/anal cancer. Sexual Med Rev. (2019) 7:202–22. doi: 10.1016/j.sxmr.2018.12.001, PMID: 30655196 PMC6445765

